# Isoprenoid biosynthesis regulation in poplars by methylerythritol phosphate and mevalonic acid pathways

**DOI:** 10.3389/fpls.2022.968780

**Published:** 2022-09-30

**Authors:** Ali Movahedi, Hui Wei, Boas Pucker, Mostafa Ghaderi-Zefrehei, Fatemeh Rasouli, Ali Kiani-Pouya, Tingbo Jiang, Qiang Zhuge, Liming Yang, Xiaohong Zhou

**Affiliations:** ^1^Key Laboratory of Forest Genetics and Biotechnology, Ministry of Education, Co-Innovation Center for Sustainable Forestry in Southern China, College of Biology and the Environment, Nanjing Forestry University, Nanjing, China; ^2^Key Laboratory of Landscape Plant Genetics and Breeding, School of Life Sciences, Nantong University, Nantong, China; ^3^Institute of Plant Biology and BRICS, TU Braunschweig, Braunschweig, Germany; ^4^Department of Animal Science, Faculty of Agriculture, Yasouj University, Yasouj, Iran; ^5^State Key Laboratory of Molecular Plant Genetics, Shanghai Center for Plant Stress Biology, Center for Excellence in Molecular Plant Sciences, Chinese Academy of Sciences, Shanghai, China; ^6^Tasmanian Institute of Agriculture, College of Science and Engineering, University of Tasmania, Hobart, TAS, Australia; ^7^State Key Laboratory of Tree Genetics and Breeding, Northeast Forestry University, Harbin, China; ^8^State Key Laboratory of Subtropical Silviculture, Zhejiang A&F University, Hangzhou, Zhejiang, China

**Keywords:** methylerythritol phosphate pathway, mevalonic acid pathway, HMGR, DXR, isoprenoid biosynthesis, poplar

## Abstract

It is critical to develop plant isoprenoid production when dealing with human-demanded industries such as flavoring, aroma, pigment, pharmaceuticals, and biomass used for biofuels. The methylerythritol phosphate (MEP) and mevalonic acid (MVA) plant pathways contribute to the dynamic production of isoprenoid compounds. Still, the cross-talk between MVA and MEP in isoprenoid biosynthesis is not quite recognized. Regarding the rate-limiting steps in the MEP pathway through catalyzing 1-deoxy-D-xylulose5-phosphate synthase and 1-deoxy-D-xylulose5-phosphate reductoisomerase (DXR) and also the rate-limiting step in the MVA pathway through catalyzing 3-hydroxy-3-methylglutaryl-CoA reductase (HMGR), the characterization and function of *HMGR* from *Populus trichocarpa* (*PtHMGR*) were analyzed. The results indicated that *PtHMGR* overexpressors (OEs) displayed various MEP and MVA-related gene expressions compared to NT poplars. The overexpression of *PtDXR* upregulated MEP-related genes and downregulated MVA-related genes. The overexpression of *PtDXR* and *PtHMGR* affected the isoprenoid production involved in both MVA and MEP pathways. Here, results illustrated that the *PtHMGR* and *PtDXR* play significant roles in regulating MEP and MVA-related genes and derived isoprenoids. This study clarifies cross-talk between MVA and MEP pathways. It demonstrates the key functions of *HMGR* and *DXR* in this cross-talk, which significantly contribute to regulate isoprenoid biosynthesis in poplars.

## Introduction

Isoprenoids (terpenoids) present many functional and structural properties over 50,000 distinct molecules in living organisms (Thulasiram et al., 2007). Isoprenoids play vital roles in plant growth and development, as well as in membrane fluidity, photosynthesis, and respiration. They are joining with plant-pathogen and allelopathic interactions to preserve plants from pathogens and herbivores, and they are also created to draw pollinators and seed-dispersing animals. The importance of isoprenoids has been proved in rubber products, drugs, flavors, fragrances, agrochemicals, nutraceuticals, disinfectants, and pigments (Bohlmann and Keeling, 2008). A wide range of isoprenoids is involved in photosynthesis in plants, including electron transfer, quenching of excited chlorophyll triplets, light-harvesting, and energy conversion (Malkin and Niyogi, 2000). Tetrapyrrole ring derived from heme pathway with an appended isoprenoid-derived phytol chain aim chlorophylls in all reaction centers and antenna complexes to absorb light energy and transfer electrons to the reaction centers. The light-harvesting system is maintained by the other isoprenoids, which are linear or partly cyclized carotenes and their oxygenated derivatives, xanthophylls. These carotenes and xanthophylls reduce excess excitation energy through the process of light-harvesting. However, these isoprenoids are attractive in flowers and fruits (Rodriguez-Concepcion, 2010). In contrast to vertebrates synthesizing cholesterol, higher plants synthesize a complex mix of sterol lipids called phytosterols (Boutte and Grebe, 2009).

Plant isoprenoids include gibberellins (GAs), carotene, lycopene, cytokinins (CKs), strigolactones (GRs), and brassinosteroids (BRs), which are produced through methylerythritol phosphate (MEP) and mevalonic acid (MVA) pathways (van Schie et al., 2006; Xie et al., 2008; Henry et al., 2015). These pathways are involved in plant growth, development, and response to environmental stresses (Bouvier et al., 2005; Kirby and Keasling, 2009). The isopentenyl diphosphate isomerase (IDI) catalyzes the conversion of the isopentenyl diphosphate (IPP) into dimethylallyl diphosphate (DMAPP), leading to provide the basic precursors for all isoprenoid productions (Hemmerlin et al., 2012; Lu et al., 2012; Zhang et al., 2019). The IPP and DMAPP are essential for regulating MEP and MVA pathway interactions (Huchelmann et al., 2014; Liao et al., 2016). The MVA pathway reactions appear in the cytoplasm, endoplasmic reticulum (ER), and peroxisomes (Cowan et al., 1997; Roberts, 2007), producing sesquiterpenoids and sterols. The 3-hydroxy-3-methylglutaryl-coenzyme A synthase (HMGR), a rate-limiting enzyme in the MVA pathway, catalyzes 3-hydroxy-3-methylglutary-CoA (HMG-CoA) to form MVA (Cowan et al., 1997; Roberts, 2007).

On the other hand, the MEP pathway reactions appear in the chloroplast, producing carotenoids, GAs, and diterpenoids. The 1-deoxy-D-xylulose5-phosphate synthase (DXS) and 1-deoxy-D-xylulose5-phosphate reductoisomerase (DXR) are rate-limiting enzymes in the MEP pathway that catalyze the conversion of glyceraldehyde3-phosphate (G-3-P) and pyruvate (Cordoba et al., 2009; Wang et al., 2012; Yamaguchi et al., 2018; Perreca et al., 2020). Isoprenoids like phytoalexin and volatile oils play essential roles in plant growth, development, and disease resistance (Hain et al., 1993; Ren et al., 2008). Besides an extensive range of natural functions in plants, terpenoids also consider the potential for biomedical applications. Paclitaxel is one of the most effective chemotherapy agents for cancer treatment, and artemisinin is an anti-malarial drug (Kong and Tan, 2015; Kim et al., 2016).

Previous metabolic engineering studies have proposed strategies to improve the production of specific plant metabolites (Ghirardo et al., 2014; Opitz et al., 2014). In addition, it has been proved that *HMGS* overexpression upregulated carotenoid and phytosterol in tomatoes (Liao et al., 2018). The HMGR has been considered as a critical factor in the metabolic engineering of terpenoids (Aharoni et al., 2005; Dueber et al., 2009), and its overexpression in ginseng (*PgHMGR1*) increased ginsenosides content, which is a necessary pharmaceutically active component (Kim et al., 2014). Overexpression of *Salvia miltiorrhiza HMGR* significantly improves diterpenoid tanshinone contents (Dai et al., 2011; Kai et al., 2011). The first generation of transgenic tomatoes exhibited a higher phytosterol content because of the overexpression of *HMGR1* from *Arabidopsis thaliana* (*AtHMGR1*; Enfissi et al., 2005).

Further studies about the MEP pathway revealed that the dissemination of *DXR* transcript resulted in decreased pigmentation amount, while its upregulation caused the accumulation of the MEP-derived isoprenoids in plastid (Carretero-Paulet et al., 2006). The overexpression of *DXR from A*. *thaliana* caused to accumulation of the diterpene anthiolimine in *Salvia sclarea* hairy roots (Vaccaro et al., 2014). In another study, the overexpression of *DXR* from peppermint resulted in approximately 50% increased monoterpene compositions (Mahmoud and Croteau, 2001). Further studies on MVA and MEP pathways exhibited their cross-talk in metabolic intermediates through plastid membranes (Laule et al., 2003; Liao et al., 2006).

Poplars provide the raw materials for industrial and agricultural production as an economic and energy species. Its fast growth characteristics and advanced resources in artificial afforestation play a vital role in the global ecosystem (Devappa et al., 2015). This study investigates the biosynthesis of isoprenoids in poplar. The increased expression of *PtHMGR* in *Populus trichocarpa* increased the transcript levels of genes associated with MVA and MEP pathways. This work further exhibits that the overexpression of *PtDXR* in *P. trichocarpa* causes downregulating of genes related to MVA and, conversely, upregulating of genes associated with MEP. The overexpression of *PtDXR* also affects GA_3_, trans-zeatin-riboside (TZR), isopentenyl adenosine (IPA), castasterone (CS), and 6-deoxocastasterone (DCS) amounts. Taken together, *PtHMGR* and *PtDXR* genes play critical regulatory points in MEP and MVA pathways through isoprenoid productions.

## Materials and methods

### Plant materials and growth conditions

The “Nanlin 895” (*Populus deltoides*×*Populus euramericana* “Nanlin895”) plants were cultured in half-strength Murashige and Skoog (1/2 MS) medium (pH 5.8) under conditions of 24°C and 74% humidity (Movahedi et al., 2015). Subsequently, NT (Transformed WT with empty vector) and transgenic poplars were cultured in 1/2 MS under 16/8 h light/dark at 24°C for 1 month (Movahedi et al., 2018).

### *PtHMGR* gene amplification and vector construction

The total RNA was extracted from *P. trichocarpa* leaves and processed with PrimeScript™ RT Master Mix, a kind of reverse transcriptase (TaKaRa, Japan). Forward and reverse primers (Supplementary Table 1: *PtHMGR*-F and *PtHMGR*-R) were designed, and the open reading frame (ORF) of *PtHMGR* was amplified *via* PCR. The total volume of 50 μl, including 2 μl primers (10 μM), 2.0 μl cDNA (200 ng), 5.0 μl 10 × PCR buffer (Mg^2+^), 4 μl dNTPs (2.5 mM), 0.5 μl rTaq polymerase (5 U; TaKaRa, Japan) was then used for the following PCR reactions: 95°C for 7 min, 35 cycles of 95°C for 1 min, 58°C for 1 min, 72°C for 1.5 min, and 72°C for 10 min. Subsequently, the product of the *PtHMGR* gene was ligated into the pEASY-T3 vector (TransGen Biotech, China) based on blue-white spot screening, and the *PtHMGR* gene was inserted into the vector pGWB9 (Song et al., 2016) using Gateway technology (Invitrogen, United States).

### Phylogenetic analyses

The National Center for Biotechnology Information database (NCBI) has been applied to download *HMGR* from *P. trichocarpa* (*PtHMGR*: Potri.004G208500; XM_006384809) and from other 34 species to align and analyze. The alignment has been performed using Geneious Prime Ver. 2022 software (Biomatters development team) to construct a phylogenetic tree with 1,000 bootstrap replicates.

### Plant transformation

#### *PtHMGR* transgenic generation and confirmation

The CDS of *PtHMGR* was cloned in pGWB9 vector, and the *Agrobacterium tumefaciens* var. EHA105, including recombinant pGWB9-*PtHMGR,* was used to transform “Nanlin 895” poplar leaves and petioles (Movahedi et al., 2014; Zhang et al., 2017). Poplar buds were screened on differentiation MS medium supplemented with 30 μg/ml Kanamycin (Kan). Resistant buds were planted in the bud elongation MS medium containing 20 μg/ml Kan and transplanted into 1/2 MS medium, including 10 μg/ml Kan to generate resistant poplar trees (Movahedi et al., 2014). Genomic DNA has been extracted from putative transformants of one-month-old leaves grown on medium-supplemented kanamycin using TianGen kits (TianGen BioTech, China). The quality of the extracted genomic DNA (250–350 ng/μl) was determined by a BioDrop spectrophotometer (United Kingdom). PCR was then conducted using designed primers (Supplementary Table 1: CaMV35S as the forward and *PtHMGR*-R as the reverse), Easy Taq polymerase (TransGene Biotech), and 50 ng of extracted genomic DNA as a template to amplify about 2,000 bp. In addition, total RNA was extracted from leaves to produce cDNA, as mentioned above. These cDNAs were then applied to real-time quantitative PCR (qPCR; Supplementary Table 1: *PtHMGR* forward and reverse) to evaluate the *PtHMGR* expression in *PtHMGR* overexpressors (*OEs*) compared with NT poplars. Three independent technical repeats were performed to analyze *PtHMGR* expression.

#### *PtDXR* transgenic supply and confirmation

*PtDXR* transformant seedlings were provided from Nanjing Xiaozhuang University through the key laboratory of quality and safety of agricultural products, which has been sequenced previously and confirmed (Xu et al., 2019), *followed by culturing under the same conditions as PtHMGR poplars. PtDXR transformants were further confirmed through PCR confirmation for T-DNA insertion.*

#### Non-transformant poplars

NT poplars were provided by transforming empty vectors into WT poplars for both PtHMGR and PtDXR transformants, followed by culturing with no medium-supplemented kanamycin to screen.

### Phenotypic properties evaluation

The 75-day-old *PtHMGR*-OEs, *PtDXR*-OEs, and NT poplars were selected to evaluate the phenotypic changes such as stem lengths (mm) and diameters (mm). *PtHMGR*-OE3 and-OE7 (Two plants) and *PtDXR*-OE1 and-OE3 (Two plants) were screened 45 times during 75 days to evaluate and compare with NT in the same conditions (Started on day 5th and continued randomly until the 75th day). All data were analyzed by GraphPad Prism 9, using ANOVA one way (Supplementary Table 2).

### Analyses of expression of MVA and MEP-related genes with qRT-PCR

Three-month-old *PtDXR*-*OEs, PtHMGR*-*OE*s, and NT (grown on soil) leaves were used to extract the total RNA. The qPCR was performed to identify MVA and MEP-related gene expressions in *PtDXR*-*OE* and *PtHMGR*-*OE*s compared to NT. We used the StepOne Plus Real-time PCR System (Applied Biosystems, United States) and SYBR Green Master Mix (Roche, Germany) to carry out qRT-PCR with the *PtActin* gene (XM-006370951) serving as the housekeeping gene standard (Zhang et al., 2013). The following conditions were used for qPCR reactions: pre-denaturation at 95°C for 10 min, 40 cycles of denaturation at 95°C for 15 s, and a chain extension at 60°C for 1 min. Three independent biological replicates were used with three technical repeats (Supplementary Table 1; *PtHMGR* and *DXR* forward and reverse).

### Quantitative detection of endogenous hormone contents

The AB Qtrap6500 mass spectrometer was used in triple four-stage rod-ion hydrazine mode. The HPLC-MS/MS method was used to quantitatively analyze GA_3_, TZR, IPA, DCS, and CS hormones. Samples were separated using Agilent 1290 HPLC with electrospray ionization as the ion source and scanned in a multi-channel detection mode. Three-month-old leaves were used for hormone extraction. Liquid nitrogen was used to grind fresh plant samples of 0.5 g, and then a mixture of 10 ml isopropanol and hydrochloric acid was added to shake for 30 min at 4°C. Next, 20 ml of dichloromethane was added to the solution, which was shaken for 30 min at 4°C. Subsequently, the collected solution was centrifuged at 13,000× g for 20 min at 4°C, and the lower organic phase was retained. Then, the organic phase was dried under nitrogen and dissolved in 400 μl methanol containing 0.1% formic acid. Finally, the collected solution was filtered through a 0.22 μm membrane and detected using HPLC-MS/MS.

The standard solutions with variable concentrations of 0.1, 0.2, 0.5, 2, 5, 20, 50, and 200 ng/ml were prepared with methanol (0.1% formic acid) as the solvent (Supplementary Figures 5–9). During plotting of the standard curve, non-linear points can be excluded. In the liquid phase, a Poroshell 120 SB-C18 column (2.1 × 150, 2.7 m) was used at a column temperature of 30°C. The mobile phase included A: B = (methanol/0.1% formic acid): (water/0.1% formic acid). Elution gradient: 0–1 min, A = 20%; 1–9 min, A = 80%; 9–10 min, A = 80%; 10–10.1 min, A = 20%; 10.1–15 min, A = 20%. The other requirements used in this experiment included 2 μl injection volume, 15 psi air curtain gas, 4,500 v spray voltage, 65 psi atomization pressure, 70 psi auxiliary pressure, and 400°C atomization temperature.

### Quantitative determination of carotenoids and lycopene contents

A sample of 1 g of poplar leaves was ground and dissolved in a mixture of 10 ml of acetone-petroleum ether (1:1). Subsequently, the supernatant was filtered and collected repeatedly. The collected solution was transferred to a liquid separation funnel and layered statically. Then, the upper organic phase was extracted through a funnel containing anhydrous sodium sulfate, and the lower layer was extracted again in petroleum ether. The collected solution was evaporated to dry with rotation at 35°C, dissolved into 1 ml dichloromethane, and filtered. Finally, the solution was filtered through a 0.45-μm filter membrane and analyzed with HPLC. In liquid phase conditions, the Waters Symmetry Shield RP18 reversed-phase chromatographic column (4.6 × 250 mm × 5 μm) was used in this study with a column temperature of 30°C. The mobile phase was a mixture of methanol, acetonitrile, and dichloromethane (methanol: acetonitrile: dichloromethane = 20: 75:5). Elution gradient: 0–30 min, mobile phase = 100%, flow rate = 1.0 ml/min. The injection volume was 10 μl. In addition, the standard curves of α-carotenoid, β-carotenoid, and lycopene were formulated as described above.

## Results

### PCR and real-time PCR confirmed the transformation of *PtHMGR*

The high similarity of achieved alignment, including lots of conserved amino acids (aa), similar domains, HMG-CoA-binding motifs (EMPVGYVQIP and TTEGCLVA), and NADPH-binding motifs (DAMGMNMV and VGTVGGGT; Ma et al., 2012), confirmed detected PtHMGR protein analytically (Supplementary Figure 1). A phylogenetic tree based on the HMGRs from various species supported the PtHMGR candidate identification (Supplementary Figure 2). The tblastn was then applied to reveal 2,614 bp *PtHMGR* on Chr04:21681480…21,684,242 with a 1,785 bp CDS. The PCR amplified 1855 bp of the *PtHMGR* from synthesized cDNA of *P. trichocarpa* confirmed the putative transgenic lines (Supplementary Figure 3A), exhibiting amplicons in PCR identification compared with NT poplar (Supplementary Figure 3B). The expression of *PtHMGR* through *PtHMGR-OEs* was significantly higher than in NT poplars (Supplementary Figure 3C), indicating successful overexpression of *PtHMGR* in poplar.

### The overexpression of *PtHMGR* and *PtDXR* regulate MVA and MEP-related genes

**Figure 1:**
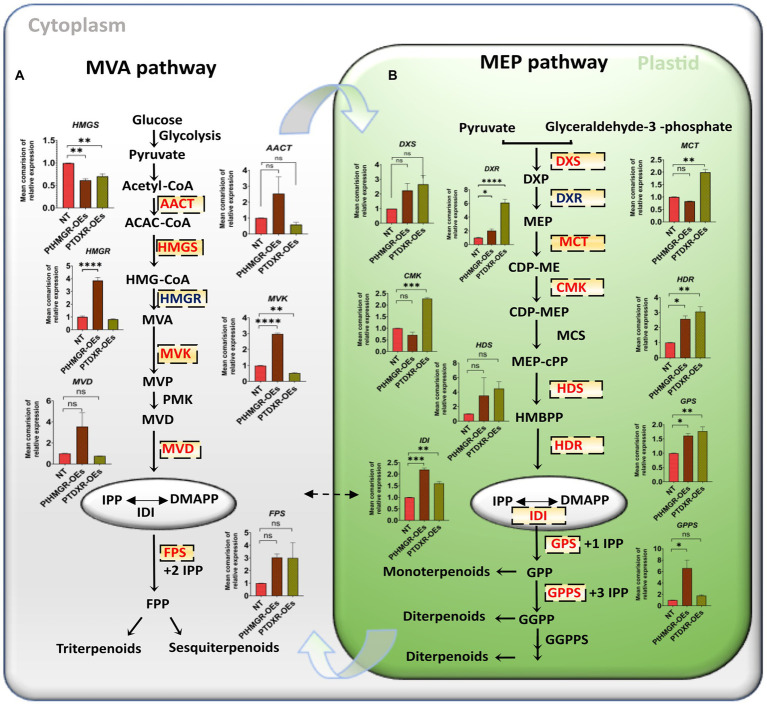
MVA and MEP-related gene expression analyses in PtHMGR-and PtDXR-OEs poplars. **(A)** Mean comparison of relative expressions of MVA related genes affected by PtHMGR-and PtDXR-OEs. **(B)** Mean comparison of relative expressions of MEP related genes affected by PtHMGR-and PtDXR-OEs; PtActin was used as an internal reference in all repeats; “ns” means not significant, *p < 0.05, ^**^p < 0.01, ^***^p < 0.001, ^****^p < 0.0001; Three independent replications were performed in each experiment.

MVA-related genes such as acetoacetyl CoA thiolase (*AACT*), mevalonate kinase (*MVK*), mevalonate5-diphosphate decarboxylase (*MVD*)*, HMGR,* and farnesyl diphosphate synthase *(FPS),* except *HMGS,* were significantly upregulated in *PtHMGR-OEs* (Figure 1A; Supplementary Figure 4A). In contrast, *PtDXR* overexpression caused upregulating of the *FPS* expression and downregulating of the *AACT*, *HMGS*, *HMGR*, *MVD*, and *MVK* expressions (Figure 1A; Supplementary Figure 4B). On the other hand, *PtHMGR*-OEs caused significantly upregulate the MEP-related genes such as *DXS*, *DXR*, 1-hydroxy-2-methyl-2-(E)-butenyl-4-diphosphate synthase (*HDS*), 1-hydroxy-2-methyl-2-(E)-butenyl-4-diphosphate reductase (*HDR*), *IDI*, geranyl pyrophosphate synthase *(GPS)*, and geranyl diphosphate synthase (*GPPS;* Figure 1B; Supplementary Figure 4C). In contrast, *PtHMGR-OEs* caused downregulating 2-C-methyl-d-erythritol4-phosphate cytidylyltransferase (*MCT*) and 4-diphosphocytidyl-2-C-methyl-D-erythritol kinase (*CMK*). Moreover, *PtDXR* overexpression caused upregulating all MEP-related genes (Figure 1B; Supplementary Figure 4D).

### *PtHMGR* positively impacts MVA and MEP-derived carotenoid contents

**Figure 2:**
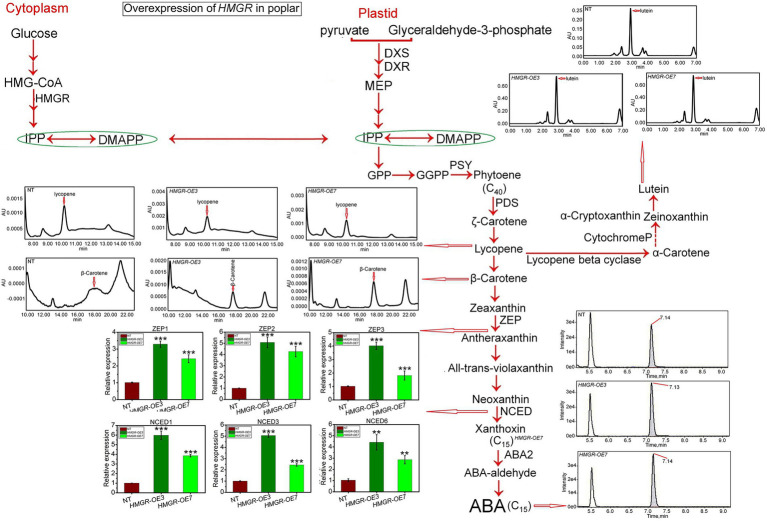
Evaluation of the effect of the overexpression of *PtHMGR* on isoprenoid production and ABA. The qRT-PCR analyses of the expression levels of ABA synthesis-related genes and HPLC-MS/MS analyses of the contents of ABA, β-carotene, lycopene, and lutein in *PtHMGR*-OEs. The expression levels of the ABA synthesis-related *ZEP* and *NCED* genes family are shown. Bars represent mean ± SD (*n* = 3); Stars reveal significant differences, ^**^p < 0.01, ^***^p < 0.001; Experiments were performed in triplicates.

β-carotene is a carotenoid synthesis that has been broadly used in the industrial composition of pharmaceuticals and as food colorants, animal supplies additives, and nutraceuticals. In addition, lycopene is a precursor of β-carotene, referring to C40 terpenoids, and is broadly found in various plants, particularly vegetables and fruits. It has been shown that MVA and MEP pathways directly influence the biosynthesis production of lycopene (Wei et al., 2018; Kim et al., 2019). While one study showed that β-carotene and lutein are synthesized using intermediates from the MEP pathway (Wille et al., 2004), the other study revealed that both MVA and MEP pathways produce isoprenoids such as β-carotene and lutein (Opitz et al., 2014). HPLC-MS/MS has been applied to analyze the MVA and MEP derivatives to quantity. Our analyses revealed a slight increase in the average lycopene content through *PtHMGR*-OE3 and-OE7 transformants compared to NT (0.0013 AU) with 0.0016 AU (Figure 2). The overexpression of *HMGR* caused significantly enhanced the synthesis of β-carotene with an average of 0.00065 AU compared with NT by-0.00002 AU (Figure 2). Results also exhibited a significant increase in lutein content through PtHMGR-OE3 and-OE7 with an average of 0.7 AU compared with NT with 0.25 AU (Figure 2). Moreover, the abscisic acid (ABA) content has been influenced by overexpression of *HMGR* with a considerable increase with an average of 3.7e4 compared with NT with 2.7e4 (Figure 2). The ABA-related gene expressions were also calculated, and results revealed that the overexpression of *HMGR* significantly upregulated the zeaxanthin epoxidase (*ZEP*) *1*, −*2*, and −3 genes with averages of ~2.8, ~4.6, and ~ 2.9, respectively in comparing with NT poplars (Figure 2). These results also indicated meaningful upregulation through 9-cis-epoxycarotenoid dioxygenase (*NCED*) 1, *−3,* and −6 genes with averages of ~4.16, ~3.79, and ~3.4 compared with NT with an average of ~1 (Figure 2).

**Figure 3:**
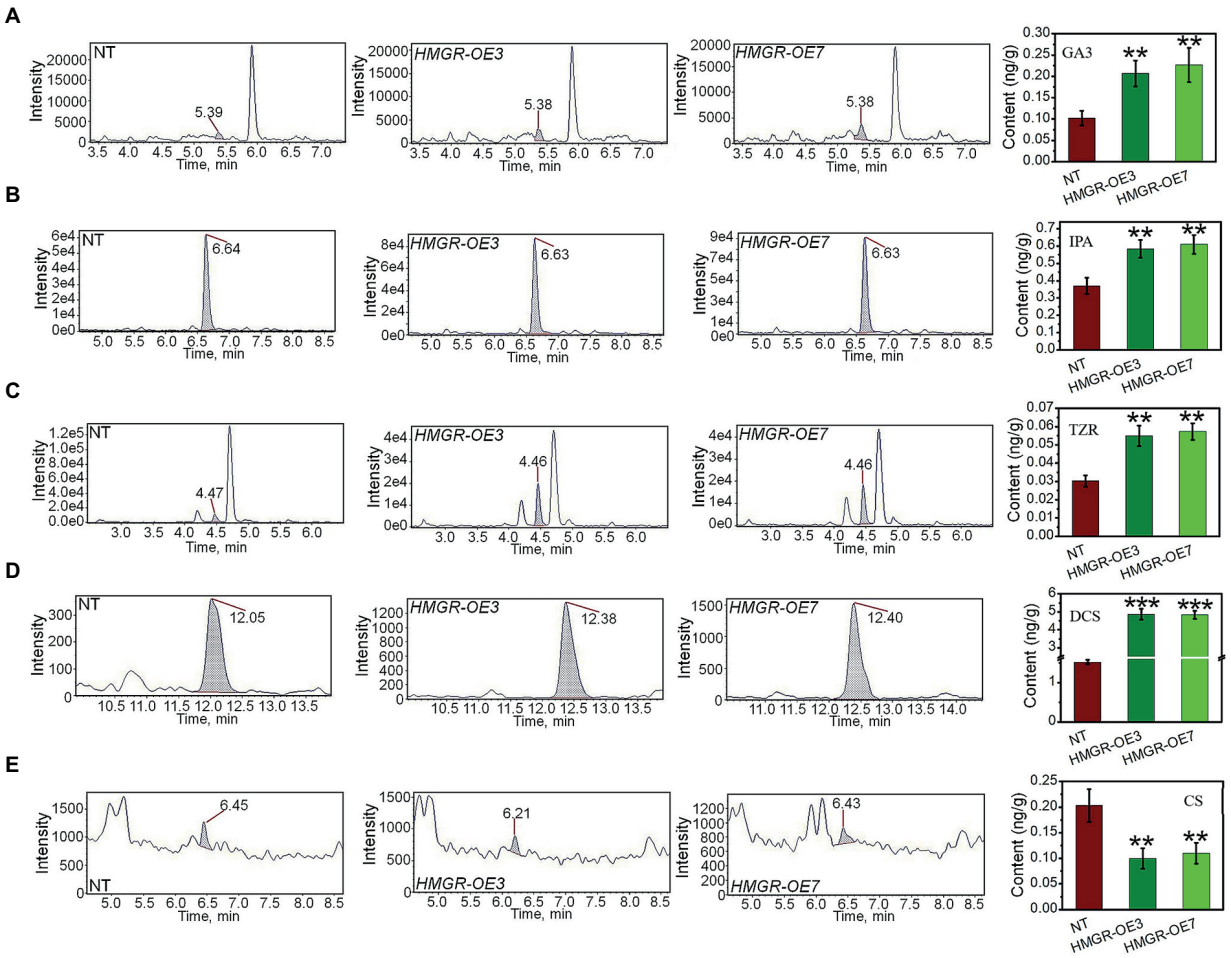
Analyzing the effect of the overexpression of *PtHMGR* on MVA and MEP-derivatives. HPLC-MS/MS analyses of the contents of GA_3_ **(A)**, IPA **(B)**, TZR **(C)**, DCS **(D)**, and CS **(E)** in *PtHMGR*-*OEs* compared to NT poplars. Bars represent mean ± SD (*n* = 3); Stars reveal significant differences, **p < 0.01, ***p < 0.001; Experiments were performed in triplicates.

In addition, the total level of GA_3_, a downstream product of MEP, increased to 0.2–0.35 ng/g in *PtHMGR*-OE lines, compared to 0.08–0.1 ng/g in NT poplars (Figure 3A). Also, the IPA content in *PtHMGR*-OEs (0.32–0.41 ng/g) is dominantly higher than that in NT (0.54–0.66 ng/g; Figure 3B). Among MVA-derived isoprenoids, the results of HPLC-MS/MS exhibited significant enhancements in the TZR content through *PtHMGR*-OEs with an average of 0.56 ng/g than NT with an average of 0.3 ng/g (Figure 3C). Surprisingly, the expression of *HMGR* caused a 3-fold increase in DCS content with an average of 5 ng/g compared with NT of about 1.5 ng/g (Figure 3D). However, *PtHMGR*-OEs negatively affected the CS content with a significant decrease compared to NT poplars (Figure 3E).

### *PtDXR* influence on MVA and MEP-derived carotenoid contents positively

**Figure 4:**
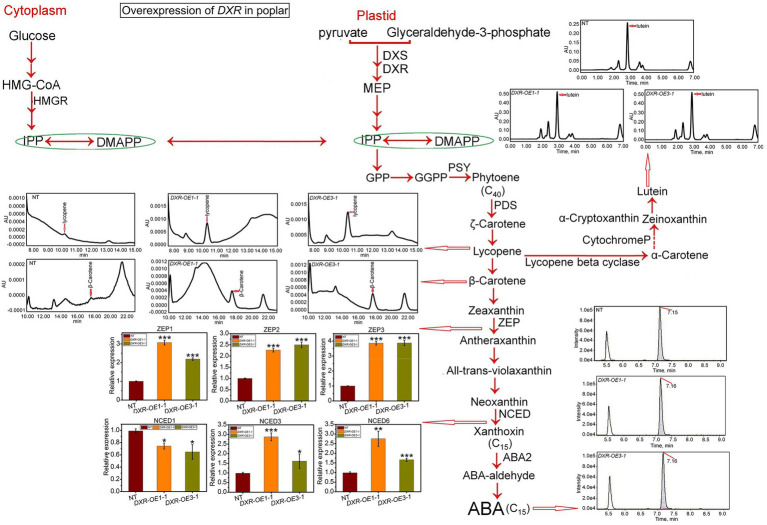
Assessment of the impact of *PtDXR* overexpression on isoprenoid and ABA biosynthesis. The qRT-PCR analyses of the expression levels of ABA synthesis-related genes and HPLC-MS/MS analyses of the contents of ABA, β-carotene, lycopene, and lutein in *PtDXR*-OEs. The expression levels of the ABA synthesis-related *ZEP* and *NCED* genes family are indicated. Bars represent mean ± SD (*n* = 3); Stars reveal significant differences, ^*^p < 0.05, ^**^p < 0.01, ^***^p < 0.001; Experiments were performed in triplicates.

*PtDXR*-OEs exhibited a significant increase in lycopene, β-carotene, and lutein contents affected by overexpression of *PtDXR* with the averages of 0.00095, 0.00037, and 0.5 AU compared to NT poplars (Figure 4). Moreover, the ABA content also has been affected by the overexpression of *PtDXR* and considerably increased more than NT poplars (Figure 4). The expression of the ABA-related gene also has been influenced by the overexpression of *PtDXR* and upregulated *NCED* genes, except for *NCED1*, which has been downregulated (Figure 4). These analyses further exhibited the positive effect of the overexpression of *PtDXR* on a significant increase in the expression of *ZEP1*, *2*, and *3* (Figure 4).

**Figure 5:**
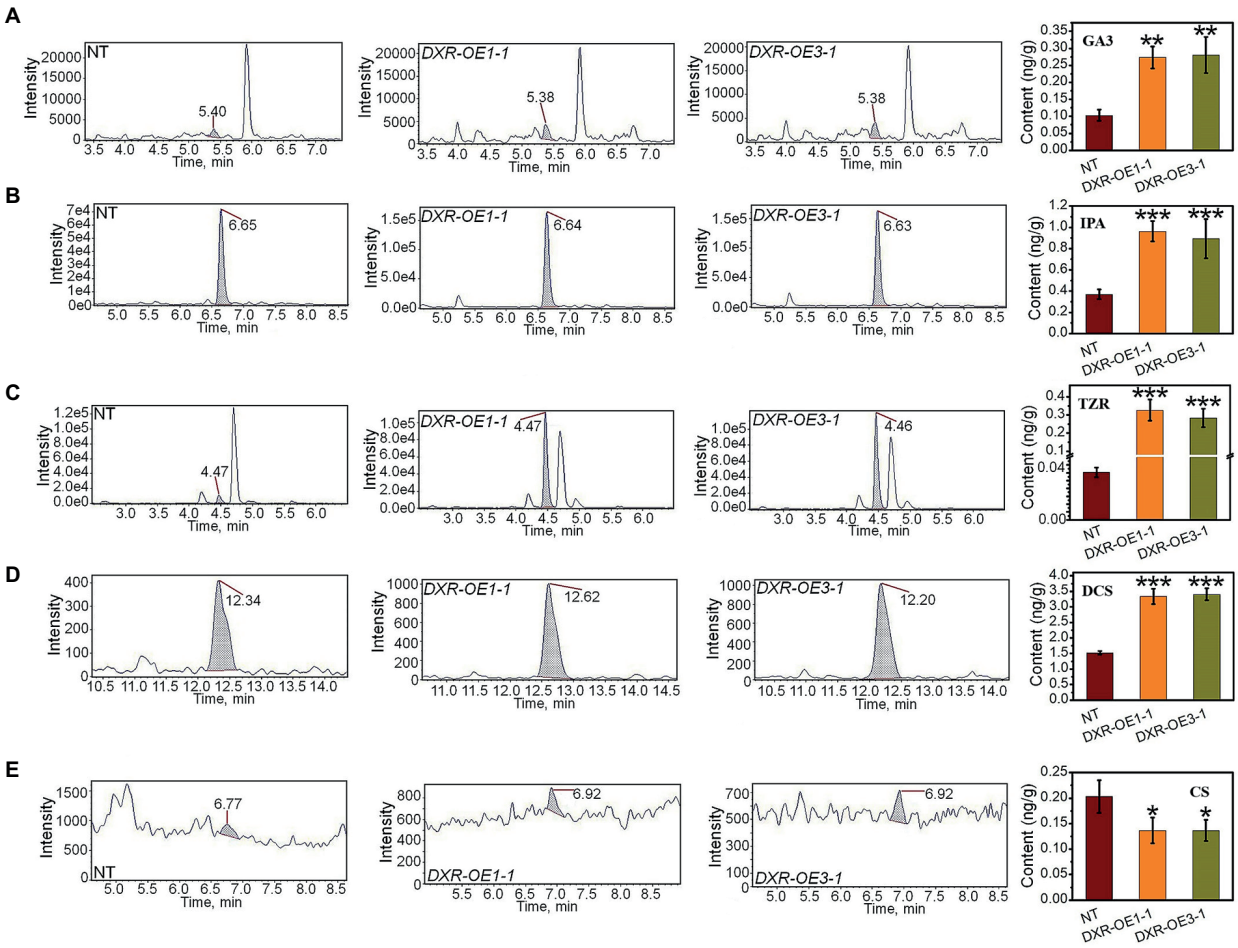
Analyzing the effect of the overexpression of *PtDXR* on MVA and MEP-derivatives. HPLC-MS/MS analyses of the contents of GA_3_ **(A)**, IPA **(B)**, TZR **(C)**, DCS **(D)**, and CS **(E)** in *PtDXR*-*OEs* compared to NT poplars.

The further analysis of the production of GA_3_ exhibited a significant increase affected by the overexpression of *PtDXR* with an average of 0.27 ng/g compared to NT poplars (Figure 5A). Also, the overexpression of *PtDXR* caused to increase in IPA production significantly (Figure 5B). A considerable increase in TZR content also was observed, influenced by the overexpression of *PtDXR* with an average of 0.3 ng/g compared to NT with only 0.04 ng/g (Figure 5C). In addition, the overexpression of *PtDXR* caused to increase in the DCS production significantly, but adversely the CS was meaningfully decreased (Figures 5D,E).

### Pleiotropic analyzes

**Figure 6:**
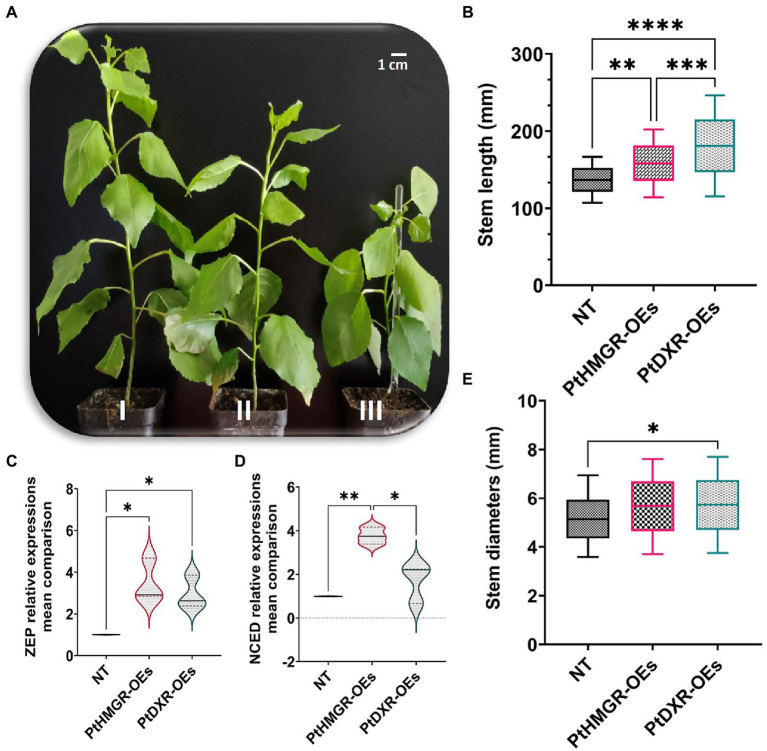
Phenotypic changes analyses resulted from the overexpression of *PtHMGR* and *PtDXR* effects on MVA and MEP pathways. (**A**, I), The *PtDXR* transgenic revealed a higher stem length than *PtHMGR*-OEs and NT poplars. (**A**, II), The *PtHMGR* transgenic presents more stem development than NT poplar. (**A**, III), NT poplar was used as a control; Scale bar represents 1 cm. **(B)** The Box and Whisker mean comparison plot of stem lengths revealed significantly higher lengths of *PtDXR*-OEs than NT poplars compared with *PtHMGR*-OEs. *PtHMGR* transgenics also revealed significantly higher lengths than NT poplars. **(C,D)** The Violin mean comparison plots of *ZEP* and *NCED* relative expressions between *PtHMGR-and PtDXR*-OEs compared with NT poplars. **(E)** The Box and Whisker mean comparison plot of stem diameters revealed slightly more diameters between *PtDXR*-OEs and NT poplars. Stars reveal significant differences, ^*^p < 0.05, ^**^p < 0.01, ^***^p < 0.001, ^****^p < 0.0001.

To improve the effect of overexpression *of PtHMGR* and *PtDXR* on phenotypic changes, the stem length and stem diameter have been evaluated. Regarding the results, the overexpression of *PtHMGR* and *PtDXR* revealed positive effects on increasing the isoprenoid contents. More increase in cytokinins TZR (~0.290 ng/g) and IPA (~0.940 ng/g) affected by the overexpression of *PtDXR* in comparing with TZR (~0.054 ng/g) and IPA (~0.580 ng/g) affected by the overexpression of *PtHMGR,* resulting in significantly more developments in the stem length through *PtDXR*-OEs compared with *PtHMGR*-OEs (Figures 6A,B). Slightly more stem diameters through *PtDXR*-OEs compared with *PtHMGR*-OEs, may be resulted from increasing the expression of ABA-related genes *ZEP* (~3.40) and *NCED* (~3.93) in *PtHMGR-OEs* compared with *ZEP* (~2.70) and *NCED* (~1.57) in *PtDXR-OEs* (Figures 6C–E).

## Discussion

### The HMGR and DXR revealed crucial roles in isoprenoid biosynthesis

Several studies report that the activity of HMGR is regulated by isoprenoid outcomes when stigmasterol and cholesterol reduce the HMGR activity by 35% (Russell and Davidson, 1982). The other study revealed a downregulation of the activity of HMGR in pea by approximately 40% among those treated with ABA, while zeatin and gibberellin improved the activity of HMGR (Russell and Davidson, 1982). Moreover, the MEP pathway is the primary precursor for required plastid isoprenoids (Maurey et al., 1986; Wright et al., 2014). It has been demonstrated that volatile compounds produced by the MEP pathway are involved in protecting plants against biotic and abiotic stresses (Gershenzon and Dudareva, 2007). Additionally, promising metabolite compounds have been developed in the mint plant by modifying the expression of *DXR* (Mahmoud and Croteau, 2002).

Overexpression of *DXR* in Arabidopsis results in accumulations of isoprenoids such as tocopherols, carotenoids, and chlorophylls (Carretero-Paulet et al., 2006). Overexpression of *DXR* has also been proven to improve diterpene content in transgenic bacteria (Morrone et al., 2010). Biotic tolerance, which is important in providing pharmaceutical terpenoids by expanding the number of enzymes included in biosynthetic pathways (Kang et al., 2009; Lu et al., 2016), caused improved *DXR* expression followed by triptophenolide content in *Tripterygium wilfordii* cell culture suspension (Tong et al., 2015).

### Overexpression of *PtHMGR* and *PtDXR* regulate the transcription of MEP and MVA-related genes

It has been shown that the overexpression of *BjHMGS1* (MVA-related gene) affected the expressions of MEP-related genes and slightly increased the transcript levels of *DXS* and *DXR* in transgenic tomatoes (Liao et al., 2018). In this study, *PtHMGR*-*OE*s exhibited the upregulation of the MEP-related genes *DXS*, *DXR*, *HDS*, and *HDR* and the downregulation of *MCT* and *CMK*. Like *BjHMGS1* overexpression in tomatoes that demonstrated a significantly upregulated of the MEP-related genes *GPS* and *GPPS* (Liao et al., 2018), we also indicated that the overexpression of *PtHMGR* enhanced the *GPS*, and *GPPS* expressions, resulting in stimulating the crosstalk between IPP and DMAPP, which increased the biosynthesis of plastidial C15 and C20 isoprenoid precursors. Moreover, it has been shown that the overexpression *of HMGR* in *Ganoderma lucidum* caused to upregulate MVA related genes *FPS*, squalene synthase (*SQS*), or lanosterol synthase (*LS*), leading to develop the contents of ganoderic acid and intermediates, including squalene and lanosterol (Xu et al., 2012). Like *the effect of* overexpression of *BjHMGS1* in tomatoes, which significantly increased transcript levels of *FPS*, *SQS*, squalene epoxidase (*SQE*), and cycloartenol synthase (*CAS*; Liao et al., 2018), this study also exhibited that the overexpression of *PtHMGR* caused to upregulate the MVA related genes *AACT*, *MVK*, *FPS*, *MVD, and except HMGS. These results proved that* the MVA-related genes contribute to the biosynthesis of sesquiterpenes.

Overexpression of MEP-related gene *TwDXR* in *Tripterygium wilfordii* promoted the expressions of MVA-related genes *TwHMGS*, *TwHMGR*, *TwFPS*, and even MEP-related gene *TwGPPS* but demoted the expression of *TwDXS* (Zhang et al., 2018). The other study also reported that the overexpression of MEP-related gene *NtDXR1* in tobacco upregulated the transcript levels of eight MEP-related genes, indicating that the overexpression of *NtDXR1* led to an improved expression of MEP-related genes (Zhang et al., 2015). In *A. thaliana*, the overexpression of *DXR* revealed no influence on regulating *DXS* gene expression or enzyme accumulation, although overexpression of *DXR* promotes MEP-derived isoprenoids such as carotenoids, chlorophylls, and taxadiene (Carretero-Paulet et al., 2006). Overexpression of potato *DXS* in *A. thaliana* led to upregulate *GGPPS* and phytoene synthase (*PSY*; Henriquez et al., 2016). In this study, the overexpression of *PtDXR* upregulated the MEP-related genes. Controversy, the *PtDXR-OEs* revealed significant downregulation of MVA-related genes. This study indicated that *PtHMGR* and *PtDXR* have important influences on MEP and MVA-related gene expression.

### Overexpression of *PtHMGR* and *PtDXR* promotes the formation of GA_3_, and carotenoids in plastids and the accumulation of TZR, IPA, and DCS in the cytoplasm

HMGR is a rate-limiting enzyme in the MVA pathway of plants and plays a critical role in controlling the flow of carbon within this metabolic pathway. The upregulation of *HMGR* significantly increases isoprenoid levels in plants. The overexpression of *HMGR* has been reported in several plants caused to promote the isoprenoid levels significantly. The heterologous expression of *Hevea brasiliensis HMGR1* in tobacco increased the sterol content and accumulated intermediate metabolites (Schaller et al., 1995). In addition, *the o*verexpression of *A*. *thaliana HMGR* in *Lavandula latifolia* increased the levels of sterols in the MVA and MEP-derived monoterpenes and sesquiterpenes (Munoz-Bertomeu et al., 2007). In addition, the overexpression *of Salvia miltiorrhiza SmHMGR* in hairy roots increased the MEP-derived diterpene tanshinone (Kai et al., 2011). In this study, ABA synthesis-related genes (*NCED1*, *NCED3*, *NCED6*, *ZEP1*, *ZEP2*, and *ZEP3*) and the contents of GA_3_ and carotenoids were upregulated in *PtHMGR*-*OE*s. These findings suggest that the overexpression *of HMGR* may indirectly affect the biosynthesis of MEP-derived isoprenoids, including GA_3_ and carotenoids. The accumulation of MVA-derived isoprenoids, including TZR, IPA, and DCS, was significantly elevated in *PtHMGR*-*OEs*, indicating that overexpression *of PtHMGR* directly influences the biosynthesis of MVA-related isoprenoids.

DXR is the rate-limiting enzyme in the MEP pathway and is an essential regulatory step in the cytoplasmic metabolism of isoprenoid compounds (Aharoni et al., 2005). It has been shown that the overexpression of *DXR* in *Mentha piperita* promotes the synthesis of monoterpenes in the oil glands and increases the production of essential oil yield by 50% (Mahmoud and Croteau, 2001). Another study exhibited that overexpression of *DXR* from *Synechocystis* sp. strain PCC6803 in tobacco resulted in increasing the β-carotene, chlorophyll, antheraxanthin, and lutein (Hasunuma et al., 2008). In addition, it has been shown that *dxr* mutants of *A*. *thaliana* revealed a lack of GAs, ABA, and photosynthetic pigments (REF57; Xing et al., 2010) and showed pale sepals and yellow inflorescences (Xing et al., 2010). This study exhibits that the overexpression of *PtDXR* positively affects the accumulation of GA_3_ and carotenoids, besides upregulation of MEP-related genes *DXS, DXR, IDI, HDS*, *HDR*, *MCT*, *CMK*, *GPS*, and *GPPS.*

### Cross-talk exists between MVA and MEP pathways in excess of IPP and DMAPP

Although the substrates of MVA and MEP pathways differ, there are common intermediates, like IPP and DMAPP (Figure 6). Blocking only the MVA or the MEP pathway, respectively, does not entirely prevent the biosynthesis of terpenes in the cytoplasm or plastids, indicating that MVA and MEP pathway products can be transported and/or move between cell compartments (Aharoni et al., 2003, 2004; Gutensohn et al., 2013). For example, it has been shown that the transferring of IPP from the chloroplast to the cytoplasm was observed through ^13^C labeling (Ma et al., 2017). In addition, segregation between the MVA and MEP pathways is limited and might exchange some metabolites over the plastid membrane (Laule et al., 2003). Some studies have applied the clustered, regularly interspaced short palindromic repeats (CRISPR) technology to reconstruct the lycopene synthesis pathway and control the flow of carbon in the MEP and MVA pathways (Kim et al., 2016). Results showed that the expressions of MVA-related genes were reduced by 81.6%, but the lycopene yield was significantly increased. By analyzing gene expression levels and metabolic outcomes in *PtHMGR-OEs* and *PtDXR-OEs*, we discovered a correlation between MVA and MEP-related genes among the derived products, which are not restricted to cross-talk between IPP and DMAPP (Figure 6).

The overexpression of *PtDXR* affected the transcript levels of MEP-related genes and the contents of MEP-derived isoprenoids, including GA_3_ and carotenoids. The weakened expressions of MVA-related genes reduce the yields of MVA-derived isoprenoids (including CS) but increase the TZR, IPA, and DCS contents. This study hypothesizes that IPP and DMAPP produced by the MEP pathway pass the cytoplasm to compensate for the lack of IPP and DMAPP of the MVA pathway to synthesize MVA-derived products.

The analysis of the overexpression of *PtHMGR* in *PtHMGR*-*OEs* exhibited higher transcript levels of MVA-related genes *AACT*, *MVK*, *FPS, MVD*, and except *HMGS* and MEP-related genes *DXS*, *DXR*, *HDS*, *HDS*, *HDR*, *IDI*, *GPS,* and *GPPS* than NT poplars, and proved that the overexpression of MVA related gene *PtHMGR* involves in regulating of both MEP and MVA pathways. These results demonstrate that cytosolic *HMGR* overexpression expanded plastidial *GPP-and GGPP*-derived products, such as GA_3_ and carotenoids, through cross-talk between MVA and MEP pathways. These results illustrate that regulation in the expression of MEP and MVA-related genes affects the MVA and MEP-derived isoprenoids.

The advanced insights in regulating MVA and MEP pathways in poplars improve the knowledge about these pathways in Arabidopsis, tomato, and rice. Altogether, these results discover that manipulating *PtDXR and PtHMGR* is a novel strategy to control poplar isoprenoids.

### The overexpression of *PtHMGR* and *PtDXR* affects cross-talk between MVA and MEP pathways, resulting in plant growth and developments

ABA and GA_3_ have been proved that perform essential functions in cell division, shoot growth, and flower induction (Xing et al., 2016). It has also been shown that the cytokinin TZR, a variety of phytohormones, performs important functions in the development and growth processes in shoots (Sakakibara, 2006; Abul et al., 2010). This study proved that the existing cross-talk between MVA and MEP pathways would be impacted positively by the overexpression of *PtHMGR* and *PtDXR*, leading to enhanced plant growth and development.

## Conclusion

Isoprenoid compounds, which have a variety of structures and properties, play a critical role in the survival and growth of plants. In this study, *PtHMGR* showed positive effects on the accumulation of MVA-derived isoprenoids and MEP-derived substances, such as ABA, GA_3_, carotenoids, and GRs, which are essential hormones for controlling growth and stress responses. *PtDXR* also exhibited an association with regulating MVA and MEP-related genes and derived isoprenoids.

## Data availability statement

The data presented in the study are deposited in NCBI (https://www.ncbi.nlm.nih.gov/nuccore/XM_006384809.2/) repository, accession number XM_006384809.2. The original contributions presented in the study are included in the article/Supplementary material.

## Author contributions

AM and HW conceived, planned, and coordinated the project, performed data analysis, wrote the draft, and finalized the manuscript. AM supervised this project. BP validated and contributed to data analysis and curation, revised and finalized the manuscript. MG-Z, FR, AK-P, and TJ reviewed and edited the manuscript. QZ, LY, and XZ coordinated, contributed to data curation, finalized, and funded this research. All authors contributed to the article and approved the submitted version.

## Funding

This work was supported by the National Natural Science Foundation of China (nos. 32001326 and 31971682), the Zhejiang Provincial Natural Science Foundation of China (nos. LZ19C160001 and LQ21C160003), the National Key Program on Transgenic Research (2018ZX08020002), and the Talent Research Foundation of Zhejiang A&F University (no. 2019FR055).

## Conflict of interest

The authors declare that this research was conducted without any commercial or financial relationships construed as a potential conflict of interest.

## Publisher’s note

All claims expressed in this article are solely those of the authors and do not necessarily represent those of their affiliated organizations, or those of the publisher, the editors and the reviewers. Any product that may be evaluated in this article, or claim that may be made by its manufacturer, is not guaranteed or endorsed by the publisher.

## Supplementary material

The Supplementary material for this article can be found online at: https://www.frontiersin.org/articles/10.3389/fpls.2022.968780/full#supplementary-material

SUPPLEMENTARY FIGURE 1 Amino acid sequences alignment of PtHMGR protein and other known HMGR proteins. The high similarities between *Populus* species and the other species are exhibited. The name of species and also their accession numbers are presented. The HMG-CoA and NADPH binding domains are indicated in red rectangular.

SUPPLEMENTARY FIGURE 2 Construction of a phylogenetic tree based on the HMGR sequences of various species. *HMGR* has been isolated from 35 species via blast on NCBI and aligned, followed by a phylogenetic tree. Results reveal a highly conserved *HMGR* through all the species. The gray rectangular shows similar *HMGR1* via *Populus* species located on one root, followed by a red dashed rectangle to show the organisms and accession numbers. The names of organisms and corresponding accession numbers are presented.

SUPPLEMENTARY FIGURE 3 Confirming of transformation of *PtHMGR*-*OEs*. **(A)** Schematic of constructed cassette transformed into the *PtHMGR* transgenic poplars. **(B)** PCR identification of *PtHMGR* in *PtHMGR*-*OEs* and NT poplars. Lane M: 15K molecular mass marker (TransGen, China); lane 1: genome DNA from NT as negative control; lanes 2–9: genome DNA from *PtHMGR-OE* lines. **(C)** qRT-PCR identifies the transcript levels of *PtHMGR* in *PtHMGR*-*OEs* and NT poplars. Three independent experiments were performed; Stars reveal significant differences, *p < 0.05, **p < 0.01, ***p < 0.001.

SUPPLEMENTARY FIGURE 4 MEP and MVA-related genes analyses in *PtHMGR*- and *PtDXR-OEs* poplars. **(A)** MVA-related genes *AACT*, *HMGS*, *HMGR*, *MVK*, *MVD,* and *FPS* are affected by the overexpression of *PtHMGR*. **(B)** MVA-related genes *AACT*, *HMGS*, *HMGR*, *MVK*, *MVD,* and *FPS* are affected by the overexpression of *PtDXR*. **(C)** MEP-related genes *DXS*, *MCT*, *CMK*, *HDS*, *HDR*, *IDI, GPS, GPPS, and DXR* are affected by the overexpression of *PtHMGR*. **(D)** MEP-related genes *DXS*, *DXR*, *MCT*, *CMK*, *HDS*, *HDR*, *IDI*, *GPS,* and *GPPS are* affected by the overexpression of *PtHMGR*. *PtActin* was used as an internal reference in all repeats; *p < 0.05, **p < 0.01, ***p < 0.001, ****p < 0.0001; Three independent replications were performed in this experiment.

SUPPLEMENTARY FIGURE 5 Chromatogram analyses of GA_3_ standards via HPLC-MS/MS. The chromatogram of standard GA_3_ at **(A)** 0.1, **(B)** 0.2, **(C)** 0.5, **(D)** 2, **(E)** 5, **(F)** 20, **(G)** 50, and **(H)** 200 ng/ml concentrations. **(I)** Equations for the GA_3_ standard curves.

SUPPLEMENTARY FIGURE 6 Chromatogram analyses of TZR standards via HPLC-MS/MS. The chromatogram of standard TZR at **(A)** 0.1, **(B)** 0.2, **(C)** 0.5, **(D)** 2, **(E)** 5, **(F)** 20, **(G)** 50, and **(H)** 200 ng/ml concentrations. **(I)** Equations for the TZR standard curves.

SUPPLEMENTARY FIGURE 7 Chromatogram analyses of IPA standards via HPLC-MS/MS. The chromatogram of standard IPA at **(A)** 0.2, **(B)** 0.5, **(C)** 2, **(D)** 5, **(E)** 20, **(F)** 50, and **(G)** 200 ng/ml concentrations. **(H)** Equations for the IPA standard curves.

SUPPLEMENTARY FIGURE 8 Chromatogram analyses of DCS standards via HPLC-MS/MS. The chromatogram of standard DCS at **(A)** 0.5, **(B)** 2, **(C)** 10, **(D)** 20, and **(E)** 50 ng/ml concentrations. **(F)** Equations for the DCS standard curves.

SUPPLEMENTARY FIGURE 9 Chromatogram analyses of CS standards via HPLC-MS/MS. The chromatogram of standard CS at **(A)** 0.5, **(B)** 5, **(C)** 10, **(D)** 20, and **(E)** 50 ng/ml concentrations. **(F)** Equations for the CS standard curves.

## Abbreviations:

MEP: Methylerythritol phosphate
MVA: Mevalonic acid
DXR: 1-deoxy-D-xylulose5-phosphate reductoisomerase
HGMR: 3-hydroxy-3-methylglutaryl-CoA reductase
GAs: Gibberellins
CKs: Cytokinins
GRs: Strigolactones
BRs: Brassinosteroids
IDI: Isopentenyl diphosphate isomerase
IPP: Isopentenyl diphosphate
DMAPP: Dimethylallyl diphosphate
HMG-CoA: 3-hydroxy-3-methylglutary-CoA
DXS: 1-deoxy-D-xylulose5-phosphate synthase
G-3-P: Glyceraldehyde3-phosphate
