# Supplementary figures and images for: Isoprenoid biosynthesis regulation in poplars by methylerythritol phosphate and mevalonic acid pathways
Source: Front Plant Sci. 2022 Sep 30;13:968780. doi: 10.3389/fpls.2022.968780 (PMC9562105; doi:10.3389/fpls.2022.968780)

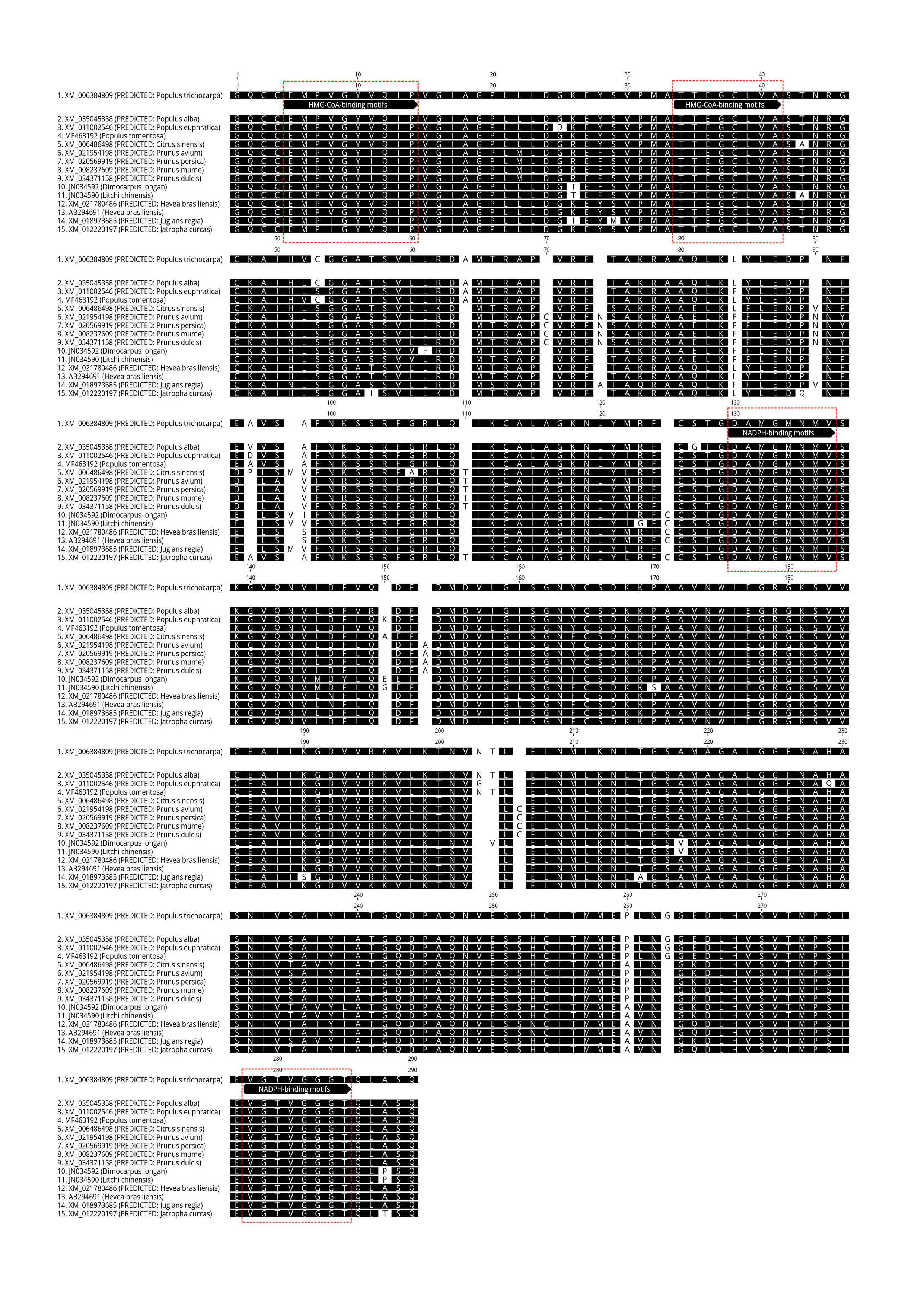

Supplement: SUPPLEMENTARY FIGURE 1 — Amino acid sequences alignment of PtHMGR protein and other known HMGR proteins. The high similarities between Populus species and the other species are exhibited. The name of species and also their accession numbers are presented. The HMG-CoA and NADPH binding domains are indicated in red rectangular. [file Image_1.TIF]

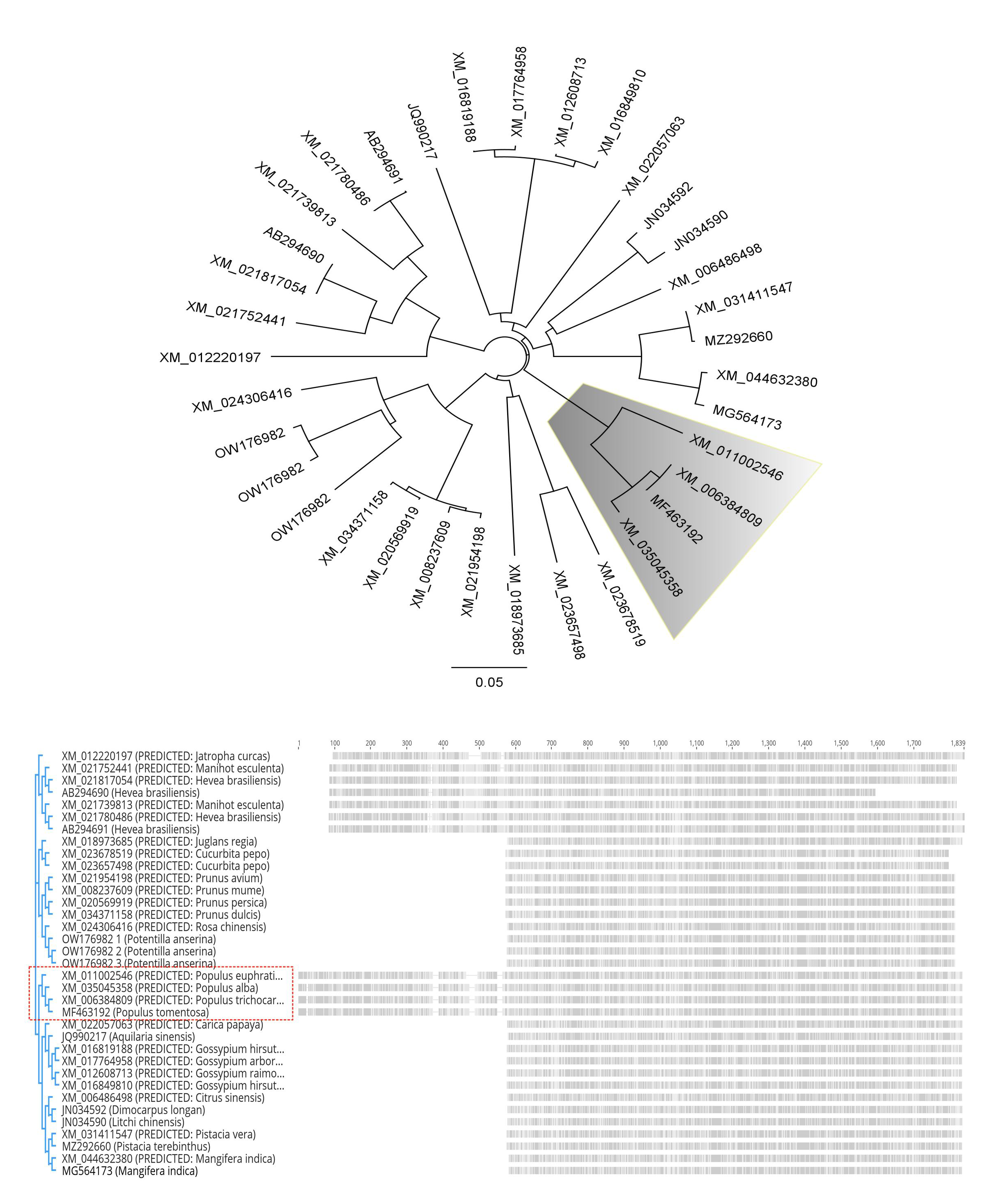

Supplement: SUPPLEMENTARY FIGURE 2 — Construction of a phylogenetic tree based on the HMGR sequences of various species. HMGR has been isolated from 35 species via blast on NCBI and aligned, followed by a phylogenetic tree. Results reveal a highly conserved HMGR through all the species. The gray rectangular shows similar HMGR1 via Populus species located on one root, followed by a red dashed rectangle to show the organisms and accession numbers. The names of organisms and corresponding accession numbers are presented. [file Image_2.TIF]

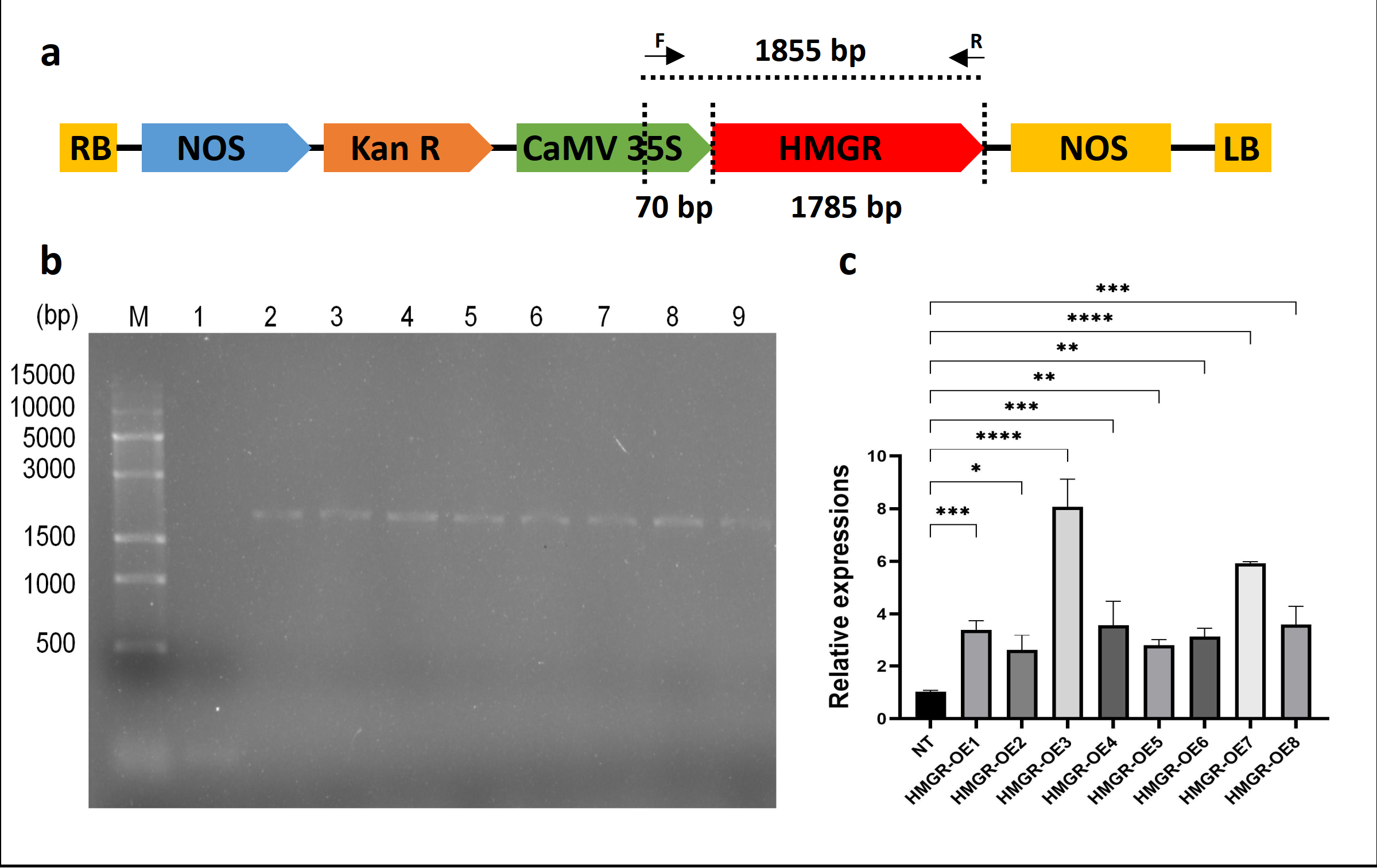

Supplement: SUPPLEMENTARY FIGURE 3 — Confirming of transformation of PtHMGR-OEs. (A) Schematic of constructed cassette transformed into the PtHMGR transgenic poplars. (B) PCR identification of PtHMGR in PtHMGR-OEs and NT poplars. Lane M: 15K molecular mass marker (TransGen, China); lane 1: genome DNA from NT as negative control; lanes 2–9: genome DNA from PtHMGR-OE lines. (C) qRT-PCR identifies the transcript levels of PtHMGR in PtHMGR-OEs and NT poplars. Three independent experiments were performed; Stars reveal significant differences, *p < 0.05, **p < 0.01, ***p < 0.001. [file Image_3.TIF]

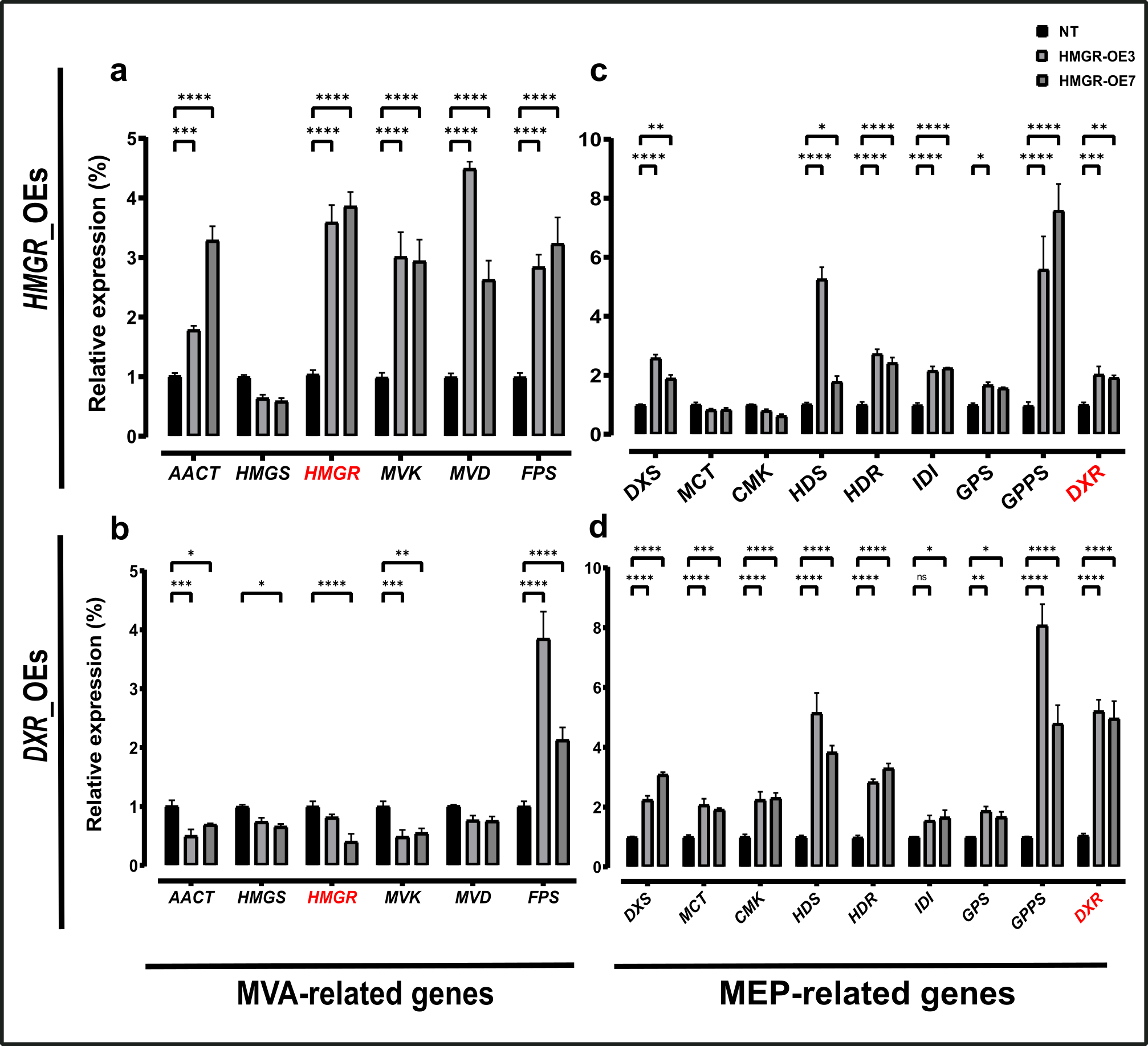

Supplement: SUPPLEMENTARY FIGURE 4 — MEP and MVA-related genes analyses in PtHMGR- and PtDXR-OEs poplars. (A) MVA-related genes AACT, HMGS, HMGR, MVK, MVD, and FPS are affected by the overexpression of PtHMGR. (B) MVA-related genes AACT, HMGS, HMGR, MVK, MVD, and FPS are affected by the overexpression of PtDXR. (C) MEP-related genes DXS, MCT, CMK, HDS, HDR, IDI, GPS, GPPS, and DXR are affected by the overexpression of PtHMGR. (D) MEP-related genes DXS, DXR, MCT, CMK, HDS, HDR, IDI, GPS, and GPPS are affected by the overexpression of PtHMGR. PtActin was used as an internal reference in all repeats; *p < 0.05, **p < 0.01, ***p < 0.001, ****p < 0.0001; Three independent replications were performed in this experiment. [file Image_4.TIF]

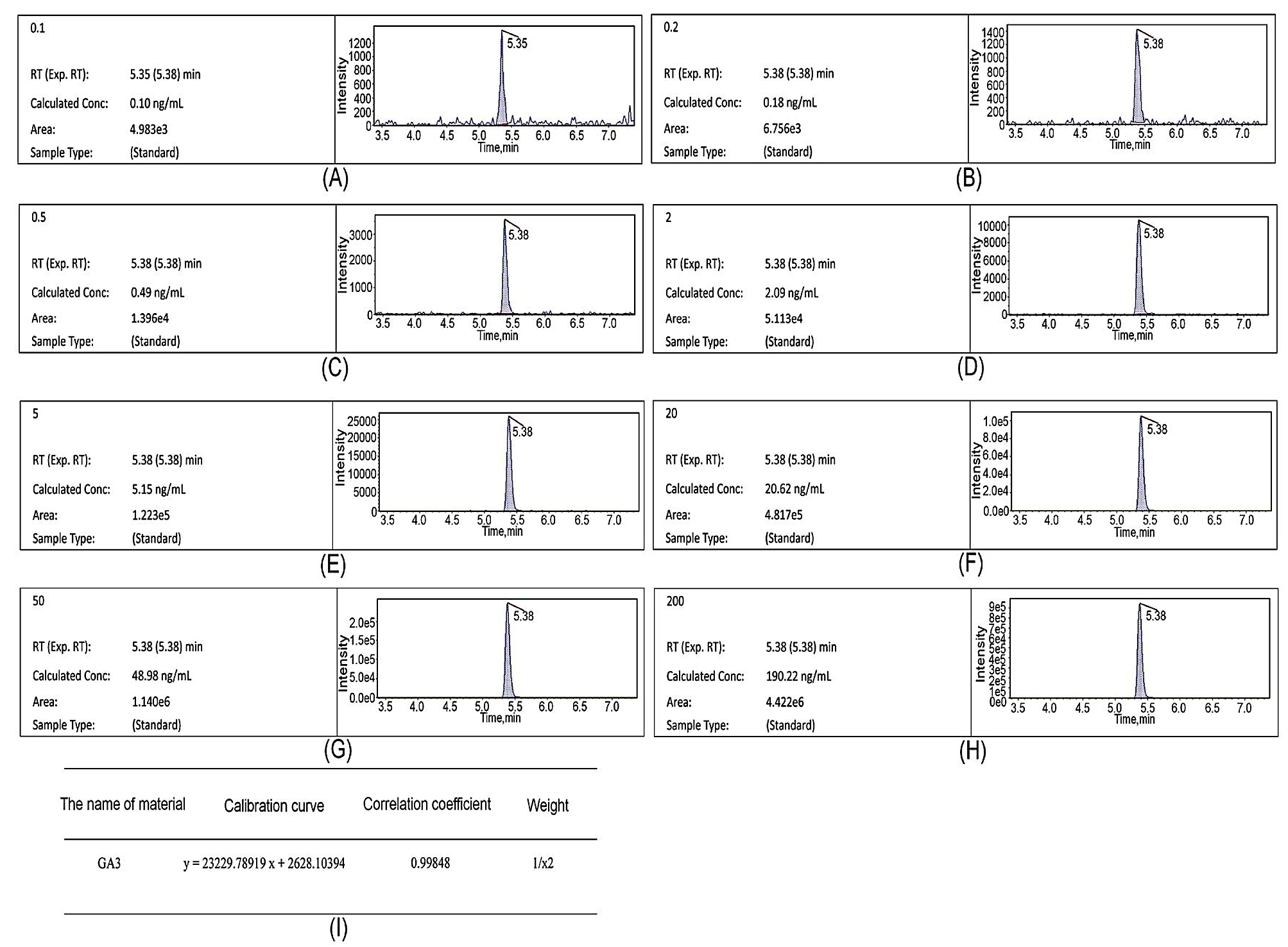

Supplement: SUPPLEMENTARY FIGURE 5 — Chromatogram analyses of GA3 standards via HPLC-MS/MS. The chromatogram of standard GA3 at (A) 0.1, (B) 0.2, (C) 0.5, (D) 2, (E) 5, (F) 20, (G) 50, and (H) 200 ng/ml concentrations. (I) Equations for the GA3 standard curves. [file Image_5.TIF]

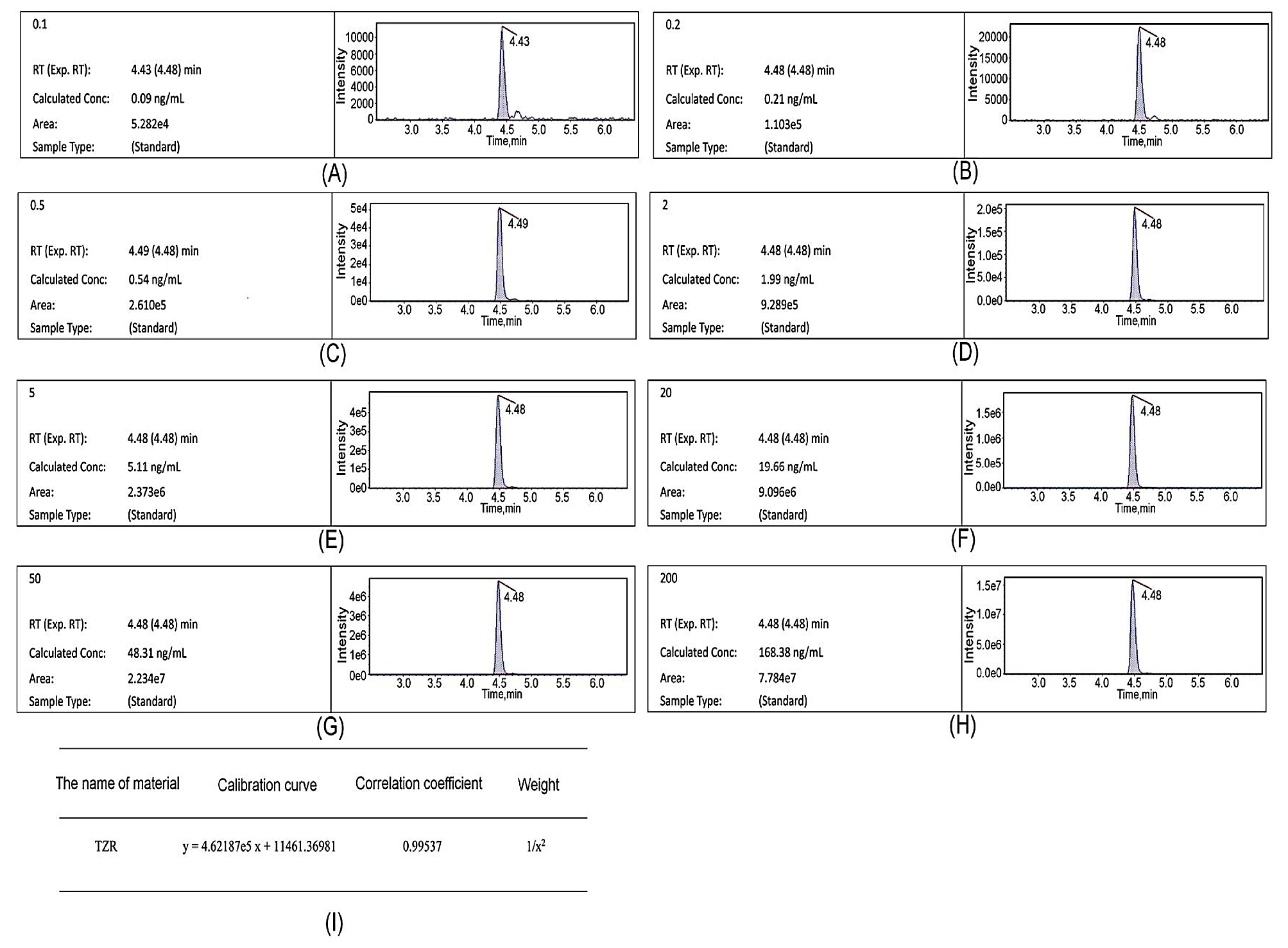

Supplement: SUPPLEMENTARY FIGURE 6 — Chromatogram analyses of TZR standards via HPLC-MS/MS. The chromatogram of standard TZR at (A) 0.1, (B) 0.2, (C) 0.5, (D) 2, (E) 5, (F) 20, (G) 50, and (H) 200 ng/ml concentrations. (I) Equations for the TZR standard curves. [file Image_6.TIF]

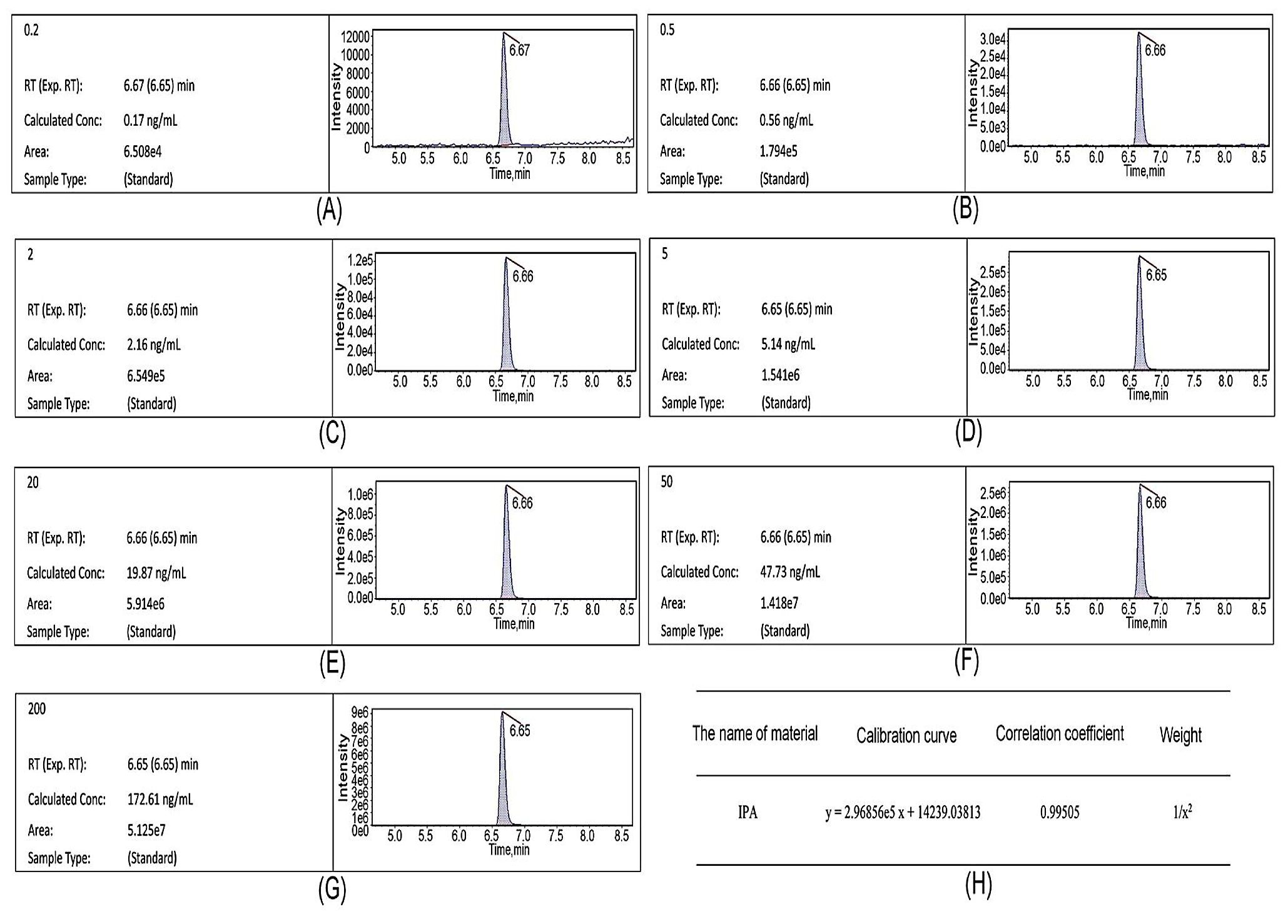

Supplement: SUPPLEMENTARY FIGURE 7 — Chromatogram analyses of IPA standards via HPLC-MS/MS. The chromatogram of standard IPA at (A) 0.2, (B) 0.5, (C) 2, (D) 5, (E) 20, (F) 50, and (G) 200 ng/ml concentrations. (H) Equations for the IPA standard curves. [file Image_7.TIF]

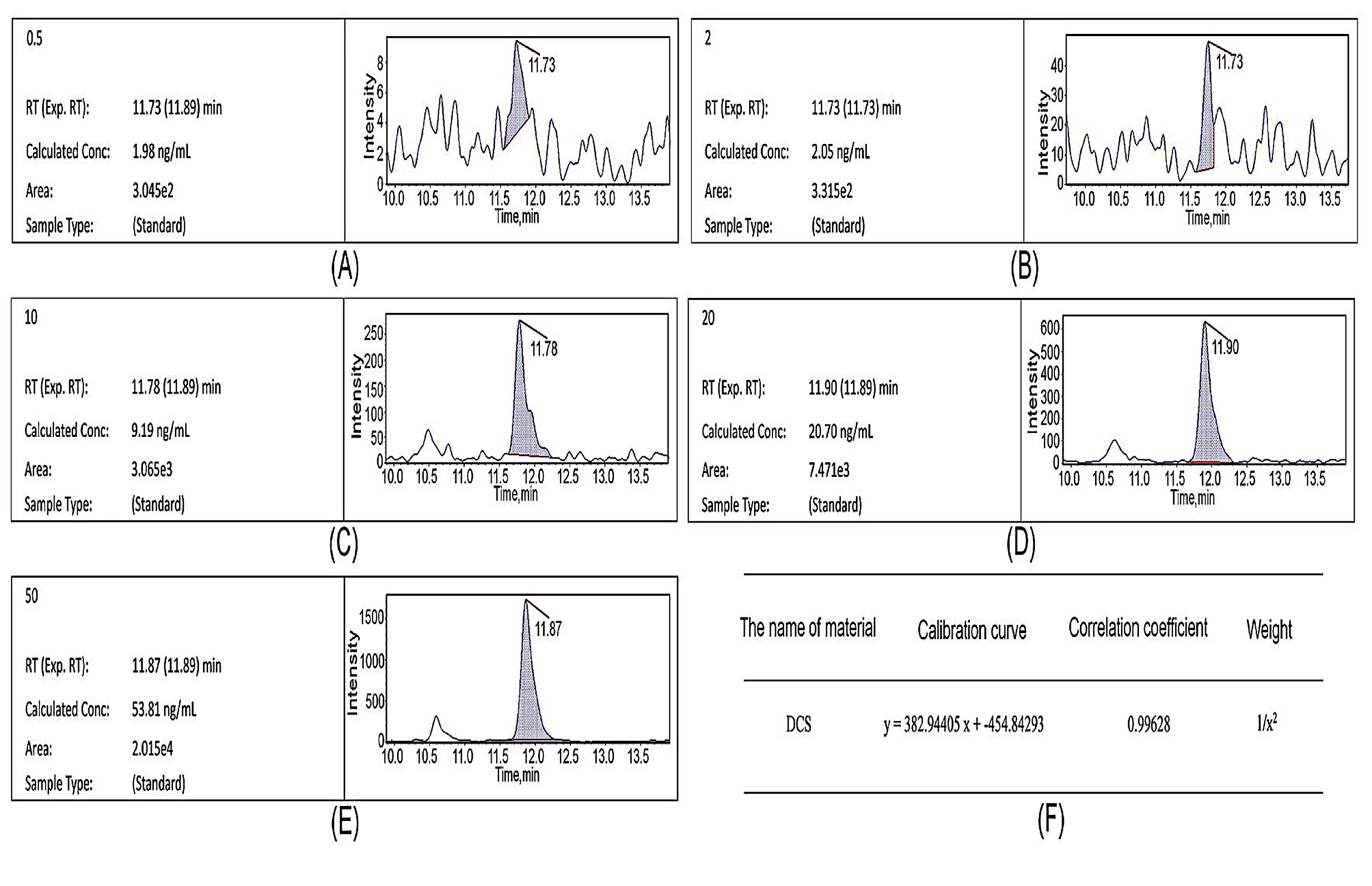

Supplement: SUPPLEMENTARY FIGURE 8 — Chromatogram analyses of DCS standards via HPLC-MS/MS. The chromatogram of standard DCS at (A) 0.5, (B) 2, (C) 10, (D) 20, and (E) 50 ng/ml concentrations. (F) Equations for the DCS standard curves. [file Image_8.TIF]

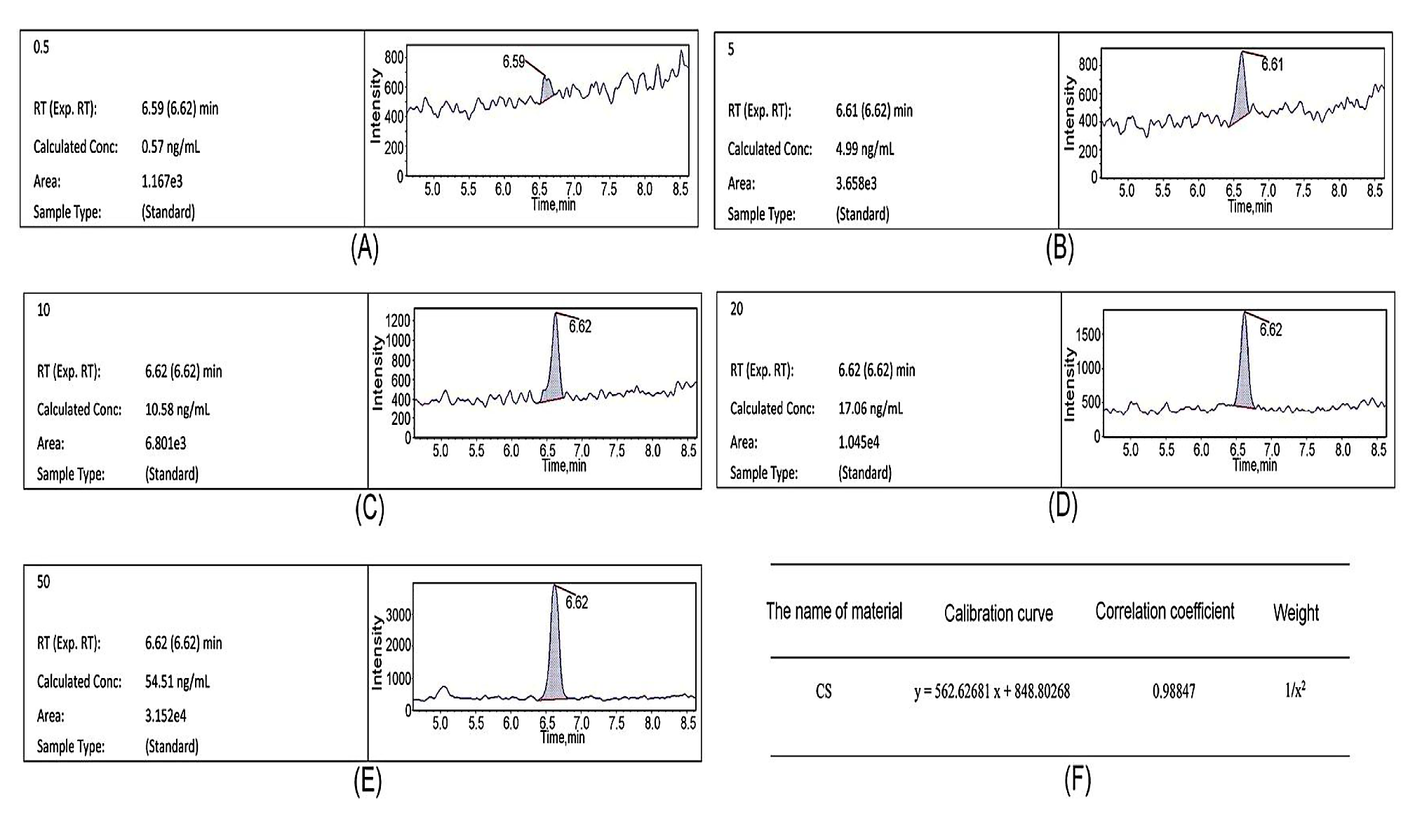

Supplement: SUPPLEMENTARY FIGURE 9 — Chromatogram analyses of CS standards via HPLC-MS/MS. The chromatogram of standard CS at (A) 0.5, (B) 5, (C) 10, (D) 20, and (E) 50 ng/ml concentrations. (F) Equations for the CS standard curves. [file Image_9.TIF]
